# Effects of Landscape Features on Bird Community in Winter Urban Parks

**DOI:** 10.3390/ani12233442

**Published:** 2022-12-06

**Authors:** Peilin Huang, Dulai Zheng, Yijing Yan, Weizhen Xu, Yujie Zhao, Ziluo Huang, Yinghong Ding, Yuxin Lin, Zhipeng Zhu, Ziru Chen, Weicong Fu

**Affiliations:** 1 College of Landscape Architecture and Art, Fujian Agriculture and Forestry University, 15 Shangxiadian Rd, Fuzhou 350002, China; 2 College of Architecture and Urban Planning, Fujian University of Technology, 33 Xuefunan Rd, Fuzhou 350118, China; 3 Engineering Research Center for Forest Park of National Forestry and Grassland Administration, 63 Xiyuangong Rd, Fuzhou 350002, China

**Keywords:** bird diversity, habitat, urbanization, urban planning

## Abstract

**Simple Summary:**

Accelerated urbanization has changed the composition of regional landscape patterns, directly affecting the composition of bird communities. This study aims to analyze the response of bird communities in urban parks in Fuzhou, China, to landscape features during winter. We selected nine urban parks in Fuzhou and investigated the distribution of their bird communities. We found that the bird community in Fuzhou is dominated by resident birds. Park area, perimeter length as well as the cover of woodland and grassland cover within the park increased with the distance from the city center. Our results showed that the park’s area, the irregularity of the park, woodland proportion, and waterbody shape have a positive effect on bird diversity. Our study is of great significance for future urban park planning and bird community diversity protection.

**Abstract:**

Urban parks, as critical components of the urban green space, have practical significance in studying the influence of landscape characteristics on birds. Nine urban parks in Fuzhou, China, were used as study objects to explore the influence of landscape features (patch, landscape, and surrounding environment indices) on bird communities. The results showed that (1) from December 2021 to February 2022, we found a total of 2874 individuals belonging to 61 species of 9 orders, 32 families, which were dominated by the birds of Passeriformes (37 species of 24 families, accounting for 89.91% of the total number of individuals) and resident birds in Fuzhou urban parks (n = 30; 85.46%); (2) The park area, park perimeter, woodland area, grassland area, and the park shape index increased as the distance to the city center increases; (3) Bird diversity responds differently to different landscape features. The total abundance of birds, the abundance of winter migrant birds, and the richness of winter migrant birds increased with the park area. And the park shape index affects positively for the the α-diversity of birds and the abundance of resident birds. Woodland proportion and waterbody shape index affected positively on the richness and α-diversity of resident birds. To promote the diversity of regional birds, it is recommended that the construction and planning of urban parks should enlarge the park area as much as possible, increase the proportion of woodland, and make shorelines more irregular. Our study could serve as a reference for the construction of biodiversity enhancements in core green areas of urban parks.

## 1. Introduction

Rapid urbanization has led to the destruction of pristine natural habitats, hence diminishing regional biodiversity [1]. Urbanization is associated with the loss or decline of bird species that existed before development, particularly ground-nesting species, habitat specialists, and those requiring extensive areas of intact habitat [2,3]. Bird species, as an integral and crucial component of urban biodiversity, are sensitive to environmental changes in their habitats and are species that are vulnerable to environmental disturbances during urbanization [4]. Although urbanization has resulted in a decline in regional biodiversity, urban green spaces can play a positive role in urban biodiversity conservation [5]. Urban green space is a significant component of the urban ecosystem and is a stable and lasting urban green environment system composed of various types of green space in the city [6]. As an important portion of urban green space, urban parks provide good habitat and food resources for birds, allowing them to cope with pressures and threats and survive in the urban matrix [2,7]. Bird diversity is frequently utilized as an essential evaluation indicator for the well-being of urban residents [8]. In addition, the selection of birds as study subjects is representative and less costly to monitor, with high research value, as confirmed by previous literature [5,9]. Therefore, reasonable monitoring of urban birds can effectively understand the urban ecological environment and is of great significance for improving residents’ well-being.

Previous studies have shown that continued urbanization affects the composition of regional landscape patterns, which in turn impacts the spatial distribution of bird communities [10]. According to the island biogeography and metapopulation theory [11,12], the urban matrix (such as building areas, transportation lands, etc.) is a physical barrier between the source and sink patches and has negative impacts on regional bird diversity [13], which is because higher environmental isolation can severely impede the spread of bird species [14]. In contrast, the presence of green space between urban matrices can substantially mitigate the isolation effect, thereby enhancing regional bird diversity [15]. Prior studies on the relationship between bird diversity and landscape features concluded that green space area, perimeter, shape index, and degree of human disturbance all have varied effects on bird diversity [16,17,18]. The patch shape of green space in parks can influence bird abundance through edge effects [19], with larger perimeter-to-area ratios enhancing the edge effect and hence having a more profound effect on bird communities. Studies of park interior patches have shown that growing fragmentation has led to the formation of different types and shapes of patches within parks. Waterbodies in urban parks play a key role in maintaining bird diversity [20], and their structure is also the main driver of forest and water bird diversity [21]. However, environmental characteristics influencing bird diversity in urban parks differ by region [22]. Therefore, given the scarcity of relevant research, it is meaningful to study the specific relationships between different landscape elements and bird diversity in urban green spaces to enhance bird diversity. 

To identify key landscape features that affect bird diversity in urban parks, we investigated the distribution of bird communities in nine urban parks within the main district of Fuzhou as the study area and quantified the key landscape features that may affect bird diversity. We aim to address the following scientific issues: (1) distribution characteristics of bird diversity in urban parks; (2) how do landscape features relate to bird diversity in urban parks, and which features have a significant impact on bird diversity; (3) how do different residence types of bird responses to landscape features, and how should these landscape features be optimized or improved to enhance the biodiversity of urban parks. We thereby provide management and planning suggestions for improving the quality of the urban ecological environment.

The following hypotheses were examined: (1) There are significant variation in species and community composition between urban parks. (2) The size of the park has significant effects on overall bird species diversity. The larger park areas may have more ecological niches that can support more species richness and abundance. (3) Woodlands and waterbodies have significant effects on winter bird diversity. Urban woodlands that have water in or near them provide habitats for an even greater variety and number of winter bird species [23].

## 2. Study Area and Methods

### 2.1. Study Area

**Figure 1:**
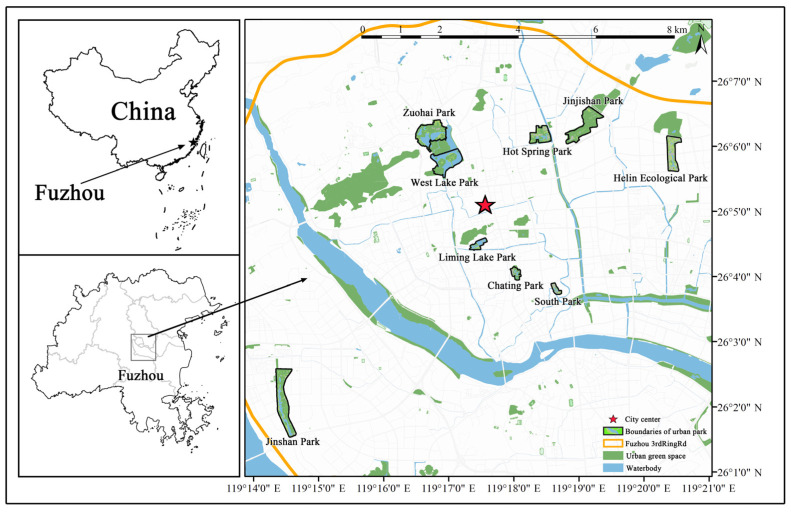
Location of 9 urban parks in Fuzhou. The Orange line represents the Fuzhou third ring road, and the red pentagram represents the location of the city center of Fuzhou (drawn by the author).

Fuzhou, commonly known as “Banyan City”, is located in eastern Fujian Province, eastern China. It is located near the downstream of the Minjiang River and coastal areas, with latitude and longitude ranging from 25°15’N–26°39’N and 118°08’E–120°31’E, respectively. The population in 2021 was 8.42 million, and the population density was 701 people/km^2^ [24]. The regional climate is a typical subtropical monsoon climate characterized by warm winters and plenty of rain and sunshine. The annual average temperature is 18–26 °C, with winter temperatures dropping down to 10–16 °C. The annual average precipitation is 1210.5 mm. Fuzhou, which belongs to the estuary basin landform, is also situated in the migratory zone from Siberia to Australia and serves as a “stopover” for migratory birds. This study selected the area within Fuzhou’s third ring road as the study area, which is the core area of Fuzhou’s main construction district and the main residential area. Our study combines a random sampling method to identify nine urban parks as the study sites (Figure 1). The selected urban parks are among Fuzhou’s typical urban parks, and they are dominated by woodlands, which act as the primary recreational places for local residents. The number of daily visitors to the selected urban parks ranges from roughly 2000 to 14,000, and the parks were similar in respect to their management statuses.

### 2.2. Bird Survey

The line transects sampling method was used in this study [25,26]. To ensure consistent sampling intensity per unit area of green space in each park, a 100 m × 50 m sample section was set up for every 2 ha of green space, and a maximum of 15 sample transects were set up in each park [27]. The bird survey was conducted in clear and windless weather from 6:00–10:00 a.m. The surveyor walked at a speed of 1–2 km/h along the pre-designed fixed sample transects, recording the species and number of birds observed and heard within 25 m to the left and right sides. Bird species hovering high in the air or flying over were not recorded. Tools used for the survey are 8 × 42 binoculars and a Nikon telephoto digital camera (Nikon COOLPIX P900s, Tokio, Japan). Bird species were identified and classified by referring to *“A Field Guide to the Birds of China”* [28]. The survey was conducted from 1 December 2021 to 20 February 2022, and each park was surveyed once a month.

### 2.3. Landscape Features

Park boundaries were plotted using the Fuzhou Urban Green Space System Plan (2016–2020) and with Google Earth 7.3.4 (Google Inc., Mountain view, CA, USA). The land cover was classified as woodland, grassland, waterbodies, sealed surface, and bare land using the unsupervised classification method (random forest classification) in ENVI 5.3 (Harris Geospatial Solutions Inc., Broomfield, CO, USA) based on GF-2 satellite images (1-m resolution, taken in February 2021). In this study, 18 major landscape features that may affect bird diversity in urban parks were selected according to the previous literature [29,30,31,32]. Landscape features were classified into three landscape types: (1) patch indices; (2) landscape indices; and (3) surrounding environment indices.

Patch indices:

(1) Waterbody area (WaA): the total area occupied by waterbody in each park, unit: ha;
(2) Waterbody proportion (WaP): % cover of waterbody in each park;
(3) Waterbody patch density (WaPD): Density of waterbody patches in each park;
  the calculated formula is: PD = NP/A

where NP is the number of patches and A is the total area of the patches. When the PD is larger, it indicates that the patches are more fragmented.

(4) Waterbody shape index (WSI): relative shape complexity of the waterbody in each park;
  the calculated formula is: SI = P / [2 × (π × A)^0.5^]

where P is the park perimeter and A is the park area. When SI = 1, the shape of the park is circular. As the shape index increases, the park shape becomes irregular and complex [5].

(5) Woodland area (WoA): the total area occupied by woodland in each park, unit: ha;
(6) Woodland proportion (WoP): % cover of woodland in each park;
(7) Grassland area (GrA): the total area occupied by grassland in each park, unit: ha;
(8) Grassland proportion (GrP): % cover of grassland in each park;
(9) Sealed surface area (SeA): the total area occupied by sealed surface in each park, unit: ha;
(10) Sealed surface proportion (SeP): % cover of sealed surface in each park;

Landscape indices:

(1) Park area (PA): the total area surrounded by the border of each park, unit: ha;
(2) Park perimeter (PP): length of the park boundary, unit: m;
(3) Park shape index (PSI): relative shape complexity of the park and calculated by the same formula as (4);

Surrounding environment indices:

(1) Distance to Fuzhou Third Ring Road (DistT): Fuzhou Third Ring Road is the boundary of the core area of the main city of Fuzhou, unit: km;
(2) Distance to city center (DistC): distance from the center of the urban park to Three Lanes and Seven Alleys, the main central area of Fuzhou and an important tourist attraction in China, unit: km;
(3) Distance to Fuzhou National Forest Park (DistF): distance to Fuzhou National Forest Park, which reflects the potential species sources of urban parks. The large size of Fuzhou National Forest Park and the low level of human exploitation is considered essential potential sources of birds in Fuzhou Park, unit: km;
(4) Woodland proportion of 500 m (WoP500): % cover of woodland within 500 m radius buffer region of the sample boundary;
(5) Sealed surface proportion of 500 m (SeP500): % cover of Sealed surface within 500 m radius buffer region of the sample boundary.

### 2.4. Analysis

Based on MacKinnon et al. (2000), we classified bird species as residents, winter migrants, summer residents, and travelers [28], and secondly we divided birds into the diet guild: insectivorous, omnivorous, herbivorous, and carnivorous based on Kissling et al. (2012) [33] (Table S2). Abundance is the total number of bird individuals in three surveys. Richness is the total number of bird species in each urban park in three surveys. Shannon diversity [34] represents the α-diversity and is calculated by the formula:

$$H^{\prime }=\overset{S}{\underset{i=1}{\sum }}P_{i}\cdot \ln P_{i}$$

*P_i_* is the ratio of the number of individuals of bird species *i* to the total number of individuals in the community. All three indexes above are commonly used for bird diversity, and they were incorporated into the subsequent analysis [35]. These analyses were performed using the “vegan” package [36] in R 4.1.2 [37].

#### 2.4.1. α-Diversity and β-Diversity

α-diversity, also known as within-habitat diversity, is the diversity within a given area or ecosystem [38]. We conducted the Chao1 estimation model [39] to predict true species richness and compare it to richness to test the completeness of bird research. The species accumulation curves [40] were calculated using the “iNEXT” package [41] to measure and predict the growth of species richness in the community as the sample size increased. This method is commonly used in biodiversity surveys to assess the adequacy of sample size and estimate community richness. We then used analysis of variance (ANOVA) [42] to analyze whether there were significant differences in overall bird alpha-diversity, richness, and abundance between different urban parks.

β-diversity was determined from the Bray–Curtis similarity matrix of the log(X + 1)-transformed abundance data [43]. To calculate differences between urban parks, a multivariate analysis of variance (PERMANOVA) [42] was performed using Bray–Curtis distances. In addition to the overall results, we also performed pairwise comparisons. To visualize patterns of variation in species composition among urban parks, we used principal coordinates analysis (PCoA) with the “metaMDS” function of the “vegan” package [36] before applying it in the Bray–Curtis distance environment. Differences in species composition between bird communities and urban parks and between urban parks were calculated using multivariate analysis of variance with 999 orderings with the “adonis” function from the “vegan” package [36]. In addition to the overall PERMANOVA results, we also compared between pairs using the “pairwise.adonis” function. The above analyses were all performed in R 4.1.2 [37].

#### 2.4.2. Effects of Landscape Features on Bird Diversity

Before analysis, we used the Shapiro–Wilk test [44] to determine whether the response variables (abundance, richness, and α-diversity) and the 18 predictor variables were in a normal distribution. The results showed that WaA (W = 0.77458, *p*-value = 0.00711), GrP (W = 0.79149, *p*-value = 0.01143), and GrA (W = 0.82898, *p*-value = 0.03253) are not normally distributed (*p*-value < 0.05). Therefore, log10 transformation was applied to these variables for standardization, and they all followed a normal distribution after transformation.

**Figure 2:**
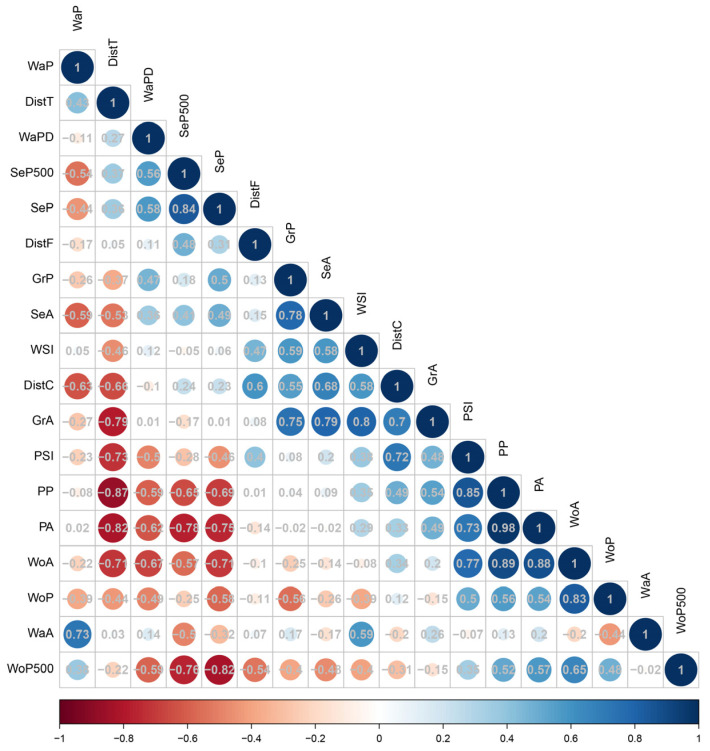
Correlation between 18 predictor variables (landscape features). The value in the circle is the correlation coefficient between the two variables. The blue circles indicate a positive correlation between the two variables, and the red circles indicate a negative correlation between the two variables.

To explore the response of urban parks to landscape characteristics variables, we conducted the following analyses. Firstly, we performed Pearson correlation analysis between bird diversity variables and landscape characteristics variables to explore the effect of single factors on bird diversity. Secondly, as variables with a strong correlation (|r| > 0.7) lead to multicollinearity [45], we utilized the Pearson correlation analysis to test for pairwise correlations between 18 environmental variables. The results showed that eleven variables—WaA, WaPD, WoA, GrA, GrP, SeA, SeP, PP, DistC, DistT, and SeP500—were found to have strong correlations with other variables (Figure 2), and the park area (PA), park shape index (PSI), waterbody proportion (WaP), waterbody shape index (WSI), woodland proportion (WoP), distance to Fuzhou National Forest Park (DistF), and woodland proportion of 500 m (WoP500) were retained. Finally, the set of variables with the strongest correlations with bird species diversity (richness, abundance, and α-Diversity) was determined using the Akaike Informativeness Criterion (AICc) [46]. The set of variables with the highest Akaike weights (Wi) was selected as the best-fit model. The above analyses were performed in R 4.1.2 [37].

## 3. Results

### 3.1. Differences in Landscape Features of Urban Parks

**Figure 3:**
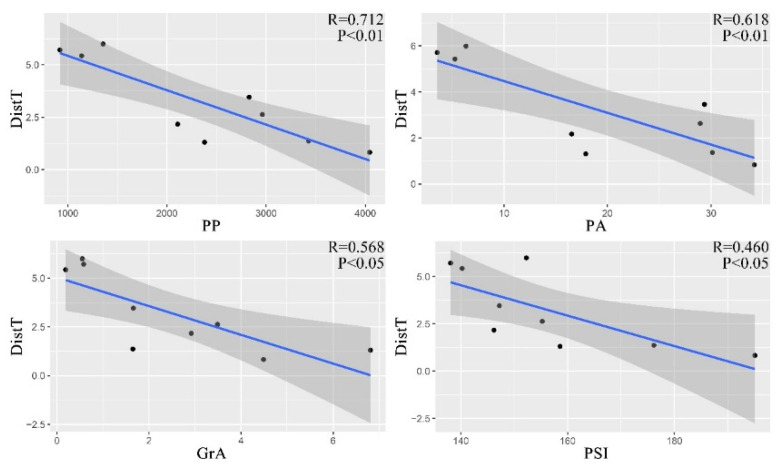
Correlation of park perimeter (PP), park area (PP), grass area (GrA), and park shape index (PSI) with distance from the third ring road (DistT). R expresses the degree of explanation of the relationship between the 2 variables, and it indicates a significant correlation between the two variables when *p* < 0.05.

The majority of landscape elements in selected Fuzhou city parks consist of woodland (about 46.43% on average), with Jinjishan Park having the highest proportion of woodland area (n = 88.32%). Helin Ecological Park has the lowest woodland area (n = 18.11%), while it is abundant in grassland landscape (n = 38.06%). Waterbody landscape, which ranked second (about 22.74% on average) among landscape elements, spanned an area from 0.03 to 15.90 ha, with the West Lake Park (n = 15.90 ha) having the most. This is followed by sealed surface (about 16.72% on average), of which Chating Park has the highest proportion (n = 23.19%). The proportion of grass landscape area varies from 3.54% to 38.06%, and the largest area proportion is in Heling Ecological Park (Table S1). The results showed that the park area, park perimeter, woodland area, grassland area, and park shape index of each park are positively correlated with the distance to the city center (Figure 3).

### 3.2. Bird Community Composition in Urban Parks

We found a total of 2874 birds belonging to 61 species of 9 orders, 32 families, dominated by the birds of Passeriformes (37 species of 24 families, accounting for 89.91% of the total number of individuals). The top eight bird species in terms of the number of individuals were light-vented bulbul (*Pycnonotus sinensis*, 590 individuals), Eurasian tree sparrow (*Passer montanus*, 407 individuals), Japanese white-eye (*Zosterops japonicus*, 349 individuals), common blackbird (*Turdus merula*, 270 individuals), yellow-browed warbler (*Phylloscopus inornatus*, 196 individuals), red-whiskered bulbul (*Pycnonotus jocosus*, 126 individuals), and Pallas’s leaf warbler (*Phylloscopus proregulus*, 90 individuals). There are three species belonging to Class II of the “List of key protected wild animals in China” [47], namely silver pheasant (*Lophura nycthemera*, 5 individuals), greater coucal (*Centropus sinensis*, 3 individuals), and hwamei (*Garrulax canorus*, 10 individuals).

**Figure 4:**
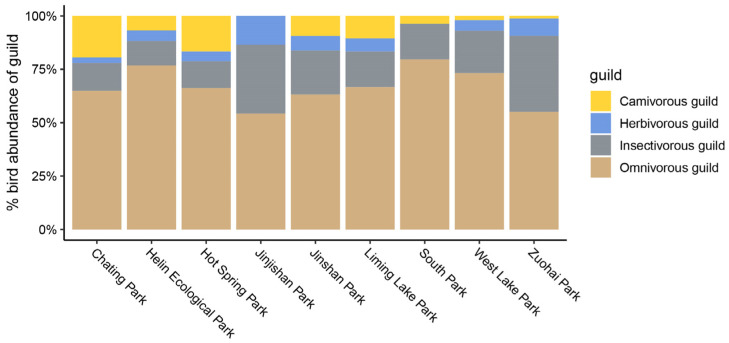
The percent-stacked bar plot represents the occurrence of the bird feeding guild abundance of 9 urban parks.

Resident birds accounted for the largest part (42 species of 27 families, accounting for 85.46% of the total number of individuals), followed by winter migrant birds (14 species of 11 families, accounting for 13.78% of the total number of individuals) and summer resident birds (3 species and 2 families, accounting for 0.35% of the total number of individuals), while only one species of traveler bird—the Asian brown flycatcher (*Muscicapa dauurica*, 126 individuals)—was recorded (Table S2). We found that the bird community in Fuzhou urban park is dominated by omnivorous, followed by insectivorous, birds (Figure 4).

### 3.3. α-Diversity and β-Diversity Varied between Different Urban Parks

**Figure 5:**
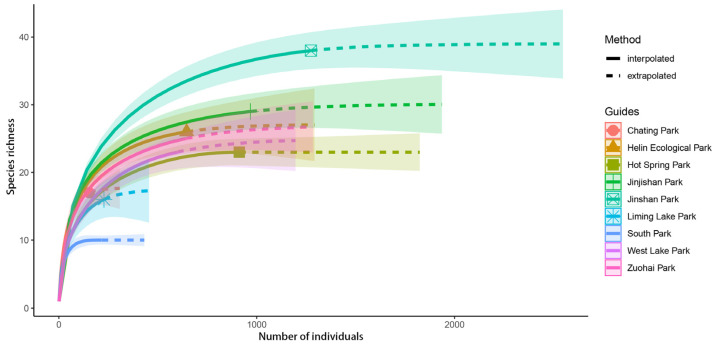
Comparison of individual-based interpolation (rarefaction) and extrapolation of species diversity in different urbanization types of parks under the multinomial model. When the curve tends to be flat, the observed number of species is gradually reasonable and more individuals will only produce fewer new species.

The results showed that as the number of individuals increases, the species accumulation curve gradually flattens out, and the number of bird species collected was nearly saturated (Figure 5). In the comparison of abundance, Jinshan Park (647 individuals) maintained a higher number of birds, followed by Jinjishan Park (529 individuals) and Hot Spring Park (456 individuals), while Chating Park (77 individuals) had the lowest number. The richness at each study site ranged from 9 to 36, with Jinshan Park (36 species) having the highest number of species and South Park (9 species) having the lowest. The survey completeness of all parks was greater than 85% (Table 1). The ANOVA analysis showed that the α-diversity (*p* < 0.001), richness (*p* < 0.001), and abundance (*p* < 0.001) were significantly different, while the results of intergroup differences in bird diversity showed no significant differences between the nine urban parks.

**Figure 6:**
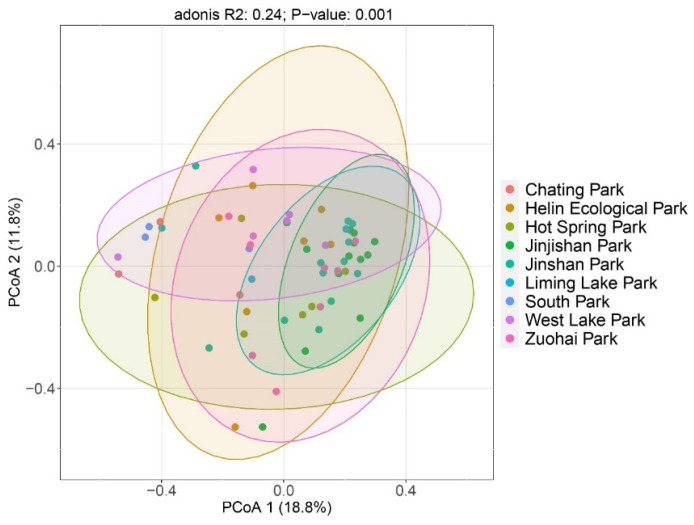
PcoA two-dimensional plot of overall birds from 9 urban parks.

The results showed that the overall bird species compositions of the selected parks were significantly different (R^2^ = 0.24, *p*-value = 0.001) (Figure 6). The result of the pairwise comparisons showed that the differences between Jinjishan Park and Chating Park (P_adj_ = 0.003), Heslin Ecological Park (P_adj_ = 0.003), Jinshan Park (P_adj_ = 0.002), South Park (P_adj_ = 0.003), Hot Spring Park (P_adj_ = 0.001), West Lake Park (P_adj_ = 0.002) and Zuohai Park (P_adj_ = 0.001); significant differences in bird species composition were found between Jinshan Park and South Park (P_adj_ = 0.036) and Hot Spring Park (P_adj_ = 0.043). There was a significant difference in bird species composition between Chating Park and Zuohai Park (P_adj_ = 0.036). And there was no significant difference (P_adj_ > 0.05) between South Park, Chating Park, Helin Ecological Park, West Lake Park, Hot Spring Park, and Liming Lake Park. Only six species have been recorded in Jinjiyama Park, namely white-crowned forktail (*Enicurus leschenaulti*), silver pheasant, white-rumped munia (Lonchura striata), orange-bellied leafbird (*Chloropsis hardwickii*), hair-crested drongo (*Dicrurus hottentottus*), rufous-capped babbler (*Stachyridopsis ruficeps*), orange-flanked bluetail (*Tarsiger cyanurus*), fire-breasted flowerpecker (*Dicaeum ignipectus*) and hawfinch (*Coccothraustes coccothraustes*).

### 3.4. Response of Bird Diversity to Landscape Features in Urban Parks

**Figure 7:**
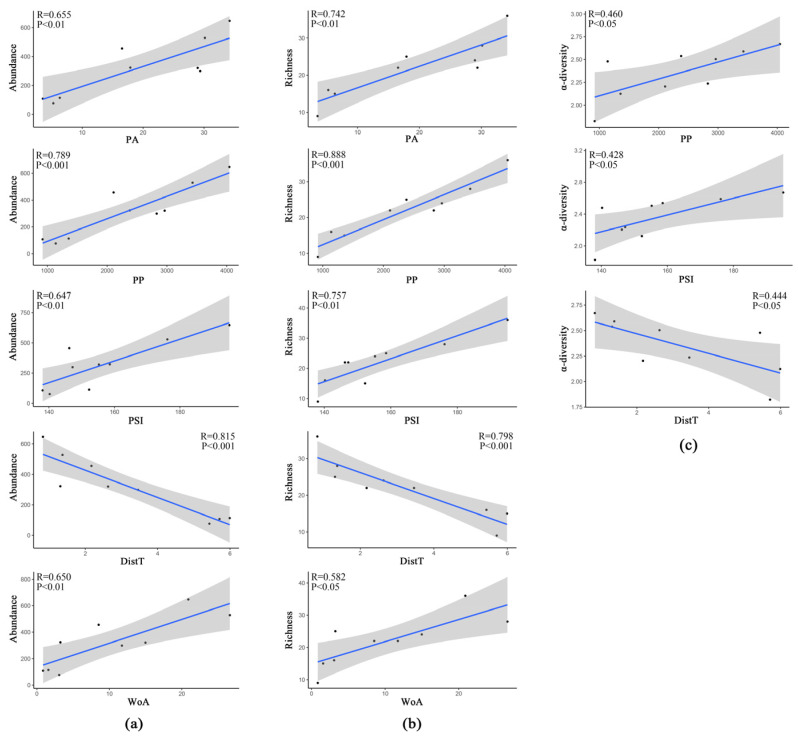
Pearson correlation of landscape features and bird diversity indicators. (**a**–**c**) are the relationships between landscape features and the overall bird species abundance, richness, and α-diversity, respectively.

The results of showed that PP, PSI and DistT were significantly correlated with overall bird abundance, richness, and α-diversity. Bird diversity increased with PP and PSI and decreased with distance to Dist T. Second, both overall bird abundance and richness were significantly and positively correlated with PA and WoA (Figure 7).

The following eight models were derived (Table S3). The models showed that PA was the best variable affecting the abundance of overall birds, the abundance of winter migrant birds, and the richness of winter migrant birds (models 1, 7, 8). PSI was the best variable affecting the α-diversity of overall birds and the abundance of resident birds (models 3, 4). PA and PSI were both the best variables affecting the richness of overall birds (model 2). Both WoP and WSI were the best variables affecting the richness of resident birds and α-diversity of resident birds (models 5, 6). Stepwise regression analysis of landscape feature indices and winter migrant bird α-diversity did not yield a linear regression in the model.

## 4. Discussion

### 4.1. Response of Bird Communities to Landscape Features in Urban Parks

In urban parks, bird diversity was significantly influenced by park landscape variables [16,48,49,50]. Our results suggested that larger parks attract more bird species, consistent with the predictions of species area theory [51,52]. Larger parks provide greater ecological space, more habitat types, and food resources for bird species, and they are conducive to the establishment of stable bird communities [30,53,54]. Apart from winter birds, park area is the most critical factor in predicting the number of breeding bird species [55]. In addition, studies have shown that nest predation rate is higher in smaller than in larger forest patches [56]. In our study, birds classified as Class II on “List of key protected wild animals in China”, such as silver pheasant and greater coucal, were only observed in Jinjishan Park and Jinshan Park. They have high requirements for habitat environment, and both of parks have a larger park area compared to other parks. Larger parks can accommodate habitat specialists that are not commonly found in smaller parks, probably because they cannot adapt well to the negative consequences of urbanization, such as landscape fragmentation and destruction of pristine natural habitats [55,57]. Most of the species in our study are residents, and their proportion of the whole community was high (68%). This is true as a general rule for urban areas in the breeding season as well [58,59]. In urban area, Resident birds have an distinct advantage over migratory birds in nest site selection since resident birds may select the few suitable nesting sites before the migratory birds arrive from their winter area [60]. At the guild level, the top five bird species in terms of the number of individuals were all omnivorous. In our observation, we found that omnivorous birds were the most abundant guild compared to other guilds and have high proportions in small parks (e.g., South Park) with high urbanization. Some omnivorous (e.g., Eurasian tree sparrow and common blackbird) birds are tolerant of human disturbance and take advantage of human waste to better adapt to urbanization [61,62,63]. Studies have shown that species numbers decline with urbanization and that highly abundant species dominate the remaining species group [2,15,64,65]. Our study also supported that omnivores extend themselves in urban areas in large numbers [66,67,68]. 

### 4.2. Response of Bird Communities to Patch Features in Urban Parks

Changing the patch layout within a park is easier to accomplish in urban park construction than expanding park size. Consistent with earlier studies [16,55,69], our findings revealed that the positive effects of land use on the number of bird species and individuals are mainly found in woodlands, and this is consistent with previous studies [70]. According to the island biogeography theory [52], the number of plant and animal species grows with the size of the woodland, which is a critical factor affecting animal habitat and an essential ecological indicator for breeding birds. We found from models 5 and 6 that the combination of woodland proportion and waterbody shape index had a highly significant positive effect on the richness of resident birds and the α-diversity of resident birds. The waterbody shape index was closely related to the “edge effect” [71]; the higher the shape indices, the longer the edge lengths. This may be due to irregular waterbody shapes which increased the “edge effect” with other land cover types, thereby providing diverse habitat conditions for birds. In subtropical regions, it is crucial that the park be able to provide adequate water resource for the bird species. In order to maintain and protect bird diversity, urban parks with limited areas should also be built with a particular amount of waterbody. Additionally, topographic treatments can be employed to generate puddles for birds. Smaller parks could also retain greater bird diversity by properly managing waterbodies and woodlands [72]. Studies have shown that the diversity, distribution, and abundance of waterbirds are largely influenced by vegetation composition and structure [73]. Our study found that grassland area has a significant positive effect on richness and abundance of birds. Grasslands in city areas often take the form of managed lawns that are constantly mowed, thus being detrimental to native specialist species (i.e., forest-, shrub-, and grassland-nesting birds) but beneficial to resident species that often occur in large numbers [7]. However, in areas with extremely cold winters, building density, density and height of trees and shrubs, length of woodland edges, etc., affect the species composition of birds since food sources are critical to individuals’ survival [23,74].

### 4.3. Implications and Prospects

Under rapid urbanization, urban parks—even small ones—are vital for birds [55]. Multiple linear regression models demonstrated that park area and park shape index are critical indicators of bird diversity in urban parks. In future urban park planning, we suggest enlarging the construction area of parks. In terms of the shape of park, except for conforming the surrounding environment, the contour boundary can be adjusted appropriately to meet the habitat and survival needs of different bird species, which is also similar to previous studies [75,76]. Furthermore, the proportion of woodland area and the shape of waterbodies are essential indicators of bird retention, which should be carefully considered in the design of green space in parks. As per our findings, the construction and management of woodland and water bodies, plus larger woodland areas within parks, are equally significant for bird diversity.

Bird diversity is influenced by a combination of environmental factors inside and outside the park. Fuzhou is a typical estuarine basin landscape with plenty of mountains, lakes, and rivers. This study focuses mostly on the interior of the park. Future research on bird environmental analysis at multiple scales is required to highlight the importance of different landscape features. Moreover, different types of environmental factors have varying effects on different groups of birds, which will be the focus of future studies on bird diversity and habitat and human wellbeing. To further explore the relationship between bird communities and the environment, future research will also consider adding more bird survey samples and monitoring bird communities in various park types for a longer period of time 

As all ecological studies do, our study has some shortcomings. For example, the sample size of the selected urban parks (n = 9) was quite small, the study was conducted only during a single winter and only in one city. Additionally, some other factors, such as noise level [77], artificial light [78], recreational activities [63], other guilds of birds [79], and vegetation habitats [80], may influence winter bird assemblage structure in urban parks.

## 5. Conclusions

Here, we investigated bird diversity in winter urban parks using the main urban area of Fuzhou as the study area. Key landscape features affecting bird diversity in urban parks were identified as potential ways to improve regional biodiversity. Management and planning recommendations for bird diversity conservation and urban park construction are informed. We found that (1) regarding landscape indices, park area (PA) and park shape index (PSI) were the main influencing factors on overall bird diversity and wintering bird diversity, and those larger park areas could provide diverse habitats for birds. (2) On the patch index, woodland proportion (WoP) and waterbody shape index (WSI) are also important for bird diversity, especially for resident birds. The interaction between woodland and water bodies can enhance regional bird diversity. To understand the annual pattern, it makes perfect sense to undertake a similar study throughout the summer. Our findings may serve as a reference for the future construction of core green spaces in urban parks to enhance biodiversity. Likewise, our findings can help improve policy and decision making in urban planning by providing normative guidelines for park construction.

## Figures and Tables

**Table 1:** Bird survey records of 9 urban parks.

No.	Response Variable	Urban Parks in Fuzhou								
		Chating Park	Helin Ecological Park	Jinjishan Park	Jinshan Park	Liming Lake Park	South Park	Hot Spring Park	West Lake Park	Zuohai Park
1	α-Diversity	2.48	2.54	2.59	2.67	2.12	1.82	2.20	2.24	2.50
2	Richness	16.00	25.00	28.00	36.00	15.00	9.00	22.00	22.00	24.00
3	Chao1	16.25	25.60	28.20	36.75	15.75	9.00	22.00	23.20	25.20
4	Completeness	0.97	0.99	1.00	0.99	0.97	1.00	1.00	0.99	0.99
5	Abundance	77	323	529	647	114	108	456	299	321

α-Diversity: The total Shannon diversity of each urban park in three surveys; Richness: the total number of bird species of each urban park in three surveys; Chao1: an estimator for species richness; Completeness: the completeness of the bird survey, calculated as Completeness = Richness/Chao1; Abundance: the total number of bird individuals in each urban park in three surveys.

## Data Availability

The data used to support the findings of this study are available from the corresponding author upon request.

## Supplementary Materials

The following supporting information can be downloaded at: https://www.mdpi.com/article/10.3390/ani12233442/s1, Table S1: Landscape features of the nine urban parks in Fuzhou, China; Table S2. Checklist and abundance of birds in the study area; Table S3: Multiple linear regression models simulating the relationships between bird diversity and landscape features.
