# Supplementary material for: Effects of Landscape Features on Bird Community in Winter Urban Parks
Source: Animals (Basel). 2022 Dec 6;12(23):3442. doi: 10.3390/ani12233442 (PMC9735841; doi:10.3390/ani12233442)
Supplement: Supplementary file 1 [file animals-12-03442-s001.zip › animals-2042410-supplementary.pdf]

Table S1. Landscape features of the 9 urban parks in Fuzhou, China.

No.	Variable	Urban Parks								
		Chating Park	Helin Ecological Park	Jinjishan Park	Jinshan Park	Liming Lake Park	South Park	Hot Spring Park	West Lake Park	Zuohai Park
1	WaA	0.78	1.56	0.03	6.25	3.25	0.48	1.76	15.90	9.55
2	WaP	14.81	8.73	0.11	18.28	51.41	13.56	10.65	54.14	32.99
3	WaPD	133.04	145.23	9.95	90.67	158.32	169.37	272.05	64.68	75.98
4	WSI	3.29	6.42	1.76	6.31	3.33	3.01	3.71	4.28	3.76
5	WoA	3.07	3.24	26.62	20.90	1.58	0.85	8.52	11.73	14.98
6	WoP	58.26	18.11	88.32	61.13	25.00	23.89	51.50	39.94	51.73
7	GrA	0.19	6.81	1.65	4.49	0.55	0.58	2.92	1.66	3.49
8	GrP	3.54	38.06	5.48	13.14	8.73	16.40	17.66	5.64	12.04
9	SeA	1.22	6.27	1.82	2.52	0.94	1.63	3.34	0.08	0.93
10	SeP	23.19	35.02	6.03	7.38	14.86	46.14	20.19	0.28	3.21
11	PA	5.26	17.90	30.14	34.19	6.32	3.54	16.54	29.37	28.96
12	PP	1140.33	2378.73	3428.29	4043.60	1356.70	921.35	2108.05	2828.61	2961.56
13	PSI	140.24	158.59	176.16	195.08	152.28	138.09	146.22	147.23	155.26
14	DistT	5.43	1.31	1.37	0.83	5.99	5.71	2.17	3.46	2.63
15	DistC	1.96	5.33	3.34	7.30	1.64	2.92	2.31	1.36	2.23
16	DistF	9.34	8.17	6.29	13.20	8.47	9.91	6.61	6.29	5.55
17	WoP500	25.48	26.62	89.95	46.89	71.51	22.62	44.85	69.39	75.68
18	SeP500	74.68	66.93	54.77	55.68	60.92	75.10	63.93	37.92	36.02

WaA, waterbody area; WaP, waterbody proportion; WaPD, waterbody patches density; WSI, waterbody shape index; WoA, woodland area; WoP, woodland proportion; GrA, grassland area; GrP, grassland proportion; SeA, sealed surface area; SeP, sealed surface proportion; PA, park area; PP, park perimeter; PSI, park shape index; DistT, distance to Fuzhou Third Ring Road; DistC, distance to city center; DistF, distance to Fuzhou National Forest Park. WoP500, woodland proportion of 500m; SeP500, sealed surface proportion of 500m.

Table S2. Checklist and abundance of birds in the study area.

No.	Species name	*Scientific name*	Family	Order	Endangerment Category	Residence types	Total numbers	Guild
1	Little Grebe	*Tachybaptus ruficollis*	*Podicedidae*	*Podicipediformes*		R	15	O
2	Common Kingfisher	*Alcedo atthis*	*Alcedinidae*	*Coraciiformes*		R	3	C
3	Pied Kingfisher	*Ceryle rudis*				R	7	O
4	Spotted Dove	*Spilopelia chinensis*	*Columbidae*	*Columbiformes*		R	2	H
5	Oriental Turtle Dove	*Streptopelia orientalis*				R	41	O
6	Common Moorhen	*Gallinula chloropus*	*Rallidae*	*Gruiformes*		R	3	O
7	White-breasted Waterhen	*Amaurornis phoenicurus*				R	11	O
8	Silver Pheasant	*Lophura nycthemera*	*Phasianidae*	*Galliformes*	Class II	R	5	O
9	Greater Coucal	*Centropus sinensis*	*Cuculidae*	*Cuculiformes*	Class II	R	3	I
10	Light-vented Bulbul	*Pycnonotus sinensis*	*Pycnonotidae*	*Passeriformes*		R	349	O
11	Red-whiskered Bulbul	*Pycnonotus jocosus*				R	4	O
12	Black Bulbul	*Hypsipetes leucocephalus*				R	8	I
13	Chestnut Bulbul	*Hemixos castanonotus*				R	2	I
14	Mountain Bulbul	*Ixos mcclellandii*				R	2	O
15	Sooty-headed Bulbul	*Pycnonotus aurigaster*				R	33	I

16	Long-tailed Shrike	*Lanius schach*	*Laniidae*			R	590	O
17	Chinese Grey Shrike	*Lanius sphenocercus*				W	5	H
18	Common Blackbird	*Turdus merula*	*Turdidae*			R	59	I
19	Pale Thrush	*Turdus pallidus*				W	12	I
20	Black-naped Oriole	*Oriolus chinensis*	*Oriolidae*			S	16	H
21	White Wagtail	*Motacilla alba*	*Motacillidae*			R	6	O
22	Olive-backed Pipit	*Anthus hodgsoni*				W	18	I
23	Scarlet Minivet	*Pericrocotus speciosus*	*Passeriformes*			R	6	O
24	Ashy Drongo	*Dicrurus leucophaeus*	*Dicruridae*			S	2	I
25	Hair-crested Drongo	*Dicrurus hottentottus*				S	47	I
26	Black-collared Starling	*Gracupica nigricollis*	*Sturnidae*			R	18	O
27	Crested Myna	*Acridotheres cristatellus*				R	13	I
28	White-cheeked Starling	*Spodiopsar cineraceus*				W	9	H
29	Yellow-browed Warbler	*Phylloscopus inornatus*	*Phylloscopidae*			W	6	H
30	Pallas's Leaf Warbler	*Phylloscopus proregulus*				W	3	I
31	Rufous-capped Babbler	*Stachyridopsis ruficeps*	*Timaliidae*			R	126	H
32	Eurasian Tree Sparrow	*Passer montanus*	*Passeridae*			R	3	I
33	White-rumped Munia	*Lonchura striata*				R	42	I
34	Hawfinch	*Coccothraustes coccothraustes*				W	2	O
35	Japanese Tit	*Parus minor*	*Paridae*			R	1	H
36	Plain Prinia	*Prinia inornata*	*Cisticolidae*			R	6	O
37	Yellow-bellied Prinia	*Prinia flaviventris*				R	10	H
38	Brownish-flanked Bush Warbler	*Horornis fortipes*	*Cettiidae*			R	2	I
39	Manchurian Bush Warbler	*Horornis borealis*				W	196	I
40	Fork-tailed Sunbird	*Aethopyga christinae*	*Nectariniidae*			R	90	I
41	Oriental Magpie Robin	*Copsychus saularis*	*Muscicapidae*			R	5	I
42	Daurian Redstart	*Phoenicurus auroreus*				W	3	O
43	Asian Brown Flycatcher	*Muscicapa dauurica*				T	3	O
44	White-crowned Forktail	*Enicurus leschenaulti*				R	46	O
45	Orange-flanked Bluetail	*Tarsiger cyanurus*				W	24	H
46	Japanese White-eye	*Zosterops japonicus*	*Zosteropidae*			R	407	O
47	Red-billed Blue Magpie	*Urocissa erythroryncha*	*Corvidae*			R	8	I
48	Azure-winged Magpie	*Cyanopica cyanus*				R	70	I
49	Japanese Grosbeak	*Eophona personata*	*Fringillidae*			W	10	O
50	Chinese Grosbeak	*Eophona migratoria*				W	270	O
51	Orange-bellied Leafbird	*Chloropsis hardwickii*	*Chloropseidae*			R	3	H
52	Common Tailorbird	*Orthotomus sutorius*	*Sylviidae*			R	1	O
53	Masked Laughingthrush	*Garrulax perspicillatus*	*Leiothrichidae*			R	37	O
54	Hwamei	*Garrulax canorus*	*Leiothrichidae*		Class II	R	2	I
55	Black-throated Bushtit	*Aegithalos concinnus*	*Aegithalidae*			R	7	O
56	Fire-breasted Flowerpecker	*Dicaeum ignipectus*	*Dicaeidae*			R	2	C
57	Black-crowned Night Heron	*Nycticorax nycticorax*	*Ardeidae*	*Pelecaniformes*		R	64	C
58	Little Egret	*Egretta garzetta*				R	41	C
59	Chinese Pond Heron	*Ardeola bacchus*				R	2	C

60	Great Egret	*Ardea alba*			W	88	C
61	Mallard	*Anas platyrhynchos*	*Anatidae*	*Anseriformes*	W	5	O

R, residents; W, winter migrants; S, summer residents; T, travelers.
Class II, Class II of the "List of key protected wild animals in China".
O, Omnivorous; C, Camivorous; I, Insectivorous; C, Camivorous.

Table S3. Multiple linear regression models simulating the relationships between bird diversity and landscape features.

No.	Model	F	R^2_{adj}	P	AICc	ΔAICc	W_i	df
Overall bird abundance								
1	ABU = 13.81PA + 55.12	16.2	0.66	0.005	119.75	0	0.34	3
Overall bird richness								
2	RIC = 0.33PA + 0.23PSI - 19.78	28.47	0.87	< 0.001	58.52	0	0.28	3
Overall bird α-diversity								
3	DIV = 0.01PSI + 0.70	6.99	0.43	0.033	5.71	0	0.28	3
Resident bird abundance								
4	ABU = 7.53PSI - 906.26	12.19	0.58	0.01	119.00	0	0.39	3
Resident bird richness								
5	RIC = 0.24WoP + 3.18WSI - 6.78	47.67	0.92	< 0.001	48.61	0	0.83	4
Resident bird α-diversity								
6	DIV = 0.01WoP + 0.12WSI + 1.10	8.67	0.66	0.017	7.65	0	0.24	4
Winter migrant bird abundance								
7	AUB = 2.37PA - 1.37	20.9	0.71	0.003	85.74	0	0.41	3
Winter migrant bird richness								
8	RIC = 0.12PA + 1.76	7.85	0.4612	0.026	40.41	0	0.28	3

R^2_{adj}: adjusted coefficient of determination.
AICc = AIC (model)+(2k (k + 1)/(n-k-1)).
W_i: Akaike weight of each model, W_i = exp (-0.5AICc)/Σexp (-0.5ΔAICc).
DIV, α-diversity of birds; RIC, bird species richness; ABU, bird abundance.
*Optimal models were selected as the final models.
